# Activation of the IGF Axis in Thyroid Cancer: Implications for Tumorigenesis and Treatment

**DOI:** 10.3390/ijms20133258

**Published:** 2019-07-02

**Authors:** Livia Manzella, Michele Massimino, Stefania Stella, Elena Tirrò, Maria Stella Pennisi, Federica Martorana, Gianmarco Motta, Silvia Rita Vitale, Adriana Puma, Chiara Romano, Sandra Di Gregorio, Marco Russo, Pasqualino Malandrino, Paolo Vigneri

**Affiliations:** 1 Department of Clinical and Experimental Medicine, University of Catania, 95123 Catania, Italy; 2 Center of Experimental Oncology and Hematology, A.O.U. Policlinico-Vittorio Emanuele, 95123 Catania, Italy; 3 Department of Medical Oncology A.O.U. Policlinico-Vittorio Emanuele, 95123 Catania, Italy; 4 Endocrinology, Department of Clinical and Experimental Medicine, Garibaldi-Nesima Medical Center, University of Catania, 95122, Italy

**Keywords:** IGF axis, thyroid cancer, therapeutic approach

## Abstract

The Insulin-like growth factor (IGF) axis is one of the best-established drivers of thyroid transformation, as thyroid cancer cells overexpress both IGF ligands and their receptors. Thyroid neoplasms encompass distinct clinical and biological entities as differentiated thyroid carcinomas (DTC)—comprising papillary (PTC) and follicular (FTC) tumors—respond to radioiodine therapy, while undifferentiated tumors—including poorly-differentiated (PDTC) or anaplastic thyroid carcinomas (ATCs)—are refractory to radioactive iodine and exhibit limited responses to chemotherapy. Thus, safe and effective treatments for the latter aggressive thyroid tumors are urgently needed. Despite a strong preclinical rationale for targeting the IGF axis in thyroid cancer, the results of the available clinical studies have been disappointing, possibly because of the crosstalk between IGF signaling and other pathways that may result in resistance to targeted agents aimed against individual components of these complex signaling networks. Based on these observations, the combinations between IGF-signaling inhibitors and other anti-tumor drugs, such as DNA damaging agents or kinase inhibitors, may represent a promising therapeutic strategy for undifferentiated thyroid carcinomas. In this review, we discuss the role of the IGF axis in thyroid tumorigenesis and also provide an update on the current knowledge of IGF-targeted combination therapies for thyroid cancer.

## 1. Introduction

Insulin-like growth factor (IGF) signaling promotes cell proliferation and differentiation in normal human tissues [1,2].

The IGF system comprises the IGF ligands 1 (IGF-1) and 2 (IGF-2) which modulate multiple biological processes by interacting with specific cell-surface receptors represented by IGF receptors I and II (IGF-IR and IGF-IIR) [3,4,5,6]. IGF binding to IGF receptors is mediated by soluble IGF binding proteins (IGFBPs), a family of six homologous molecules (IGFBP-1-6) with high binding affinity for IGF-1 and IGF-2 [2,7].

IGF-IR and IGF-IIR (IGFRs) show different structures and function. In detail, IGF-IR is a member of the receptor tyrosine kinases (RTK) and binds IGF-1 with a higher affinity than IGF-2. IGF-IR forms a tetrameric complex consisting of two alpha and two beta subunits linked by disulfide bonds through fibronectin type III (FnIII) domains. The alpha subunit localizes in the extracellular space and consists of two large homologous regions (L1 and L2) separated by a cysteine rich domain responsible for ligand binding. The beta subunit displays a trans-membrane segment and an intracellular portion containing the tyrosine kinase domain (TKD) that actively phosphorylates direct downstream targets on selected tyrosine residues [6]. Unlike IGF-IR, IGF-IIR lacks catalytic activity presenting instead a monomeric structure consisting of a large extracellular portion of 15 contiguous domains followed by a short trans-membrane region. Hence, IGF-IIR shows scavenger properties towards circulating IGF-2 [6,8].

An additional feature of the IGF axis is its crosstalk with insulin and insulin receptors isoforms A (IR-A) and B (IR-B). Indeed, the IGF system contributes to thyroid cancerogenesis via an IGF-2/IR-A autocrine loop as IGF-2 activates the IR-A isoform over-expressed in neoplastic thyrocytes, thus promoting their proliferation and suppressing apoptosis [9,10]. However, IR-A, IR-B and IGF-IR generate hybrid tetramers (IGF-IR/IR-A) (IGF-IR/IR-B), which bind both IGF-1/-2 and insulin. Upon ligand binding, these hybrid receptors promote cell proliferation and cell adhesion but inhibit programmed cell death [11,12,13].

To date, several studies have established that aberrant IGF signaling plays a critical role in cancer pathogenesis and progression. Increased expression of IGF ligands and receptors has been observed in tumors of the breast, lung, pancreas, colon, prostate, ovary, and thyroid and is usually associated with a poor prognosis [1,6,14,15].

Thyroid cancer is a frequently encountered endocrine malignancy sometimes aided by exposure to multiple environmental carcinogens favoring the generation of different molecular alterations [16,17,18,19,20]. Thyroid cancer mostly originates from the follicular epithelium and comprises differentiated (DTC), poorly differentiated (PDTC) and anaplastic (ATC) tumor histotypes. While DTCs are typically characterized by a favorable prognosis, PDTCs and ATCs have an unfavorable clinical course and are usually unresponsive to radioactive iodine because of their lack of the sodium iodide symporter (NIS) [21]. Finally, approximately 3% of all thyroid tumors are medullary thyroid carcinomas (MTC) deriving from the neoplastic transformation of neuroendocrine C-cells [21,22].

As the IGF system has been actively investigated in thyroid cancer [9,10,14,23], in this review, we will provide an update focusing on the role of the IGF axis in thyroid cancer development and treatment.

## 2. IGF Signaling in Thyroid Cancer

Deregulation of the IGF axis is emerging as a key factor promoting proliferative responses in both normal and neoplastic thyrocytes [1,10,24,25]. Indeed, while both IGF-1 and IGF-IR are expressed in benign and malignant thyroid tissues [26], Belfiore and colleagues reported that IGF-IR is more expressed in DTCs, allowing IGF-1 to exert a potent mitogenic effect [27]. These data are in agreement with an additional manuscript describing a positive correlation between serum IGF-1 levels and the risk of developing DTCs [28].

Since the IGF cascade relies on IGFBP-mediated interactions between the IGF ligands and IGF-IR, several studies have investigated if IGFBPs are involved in thyroid tumorigenesis [29,30]. Indeed, experimental data suggest that IGFBPs sequester circulating IGF-1 and IGF-2, thus limiting their IGF-IR interaction and exerting a tumor suppressor activity [1,31]. Hence, IGFBP-1 overexpression attenuates IGF-1 activity, at least in vitro [32].

**Figure 1:**
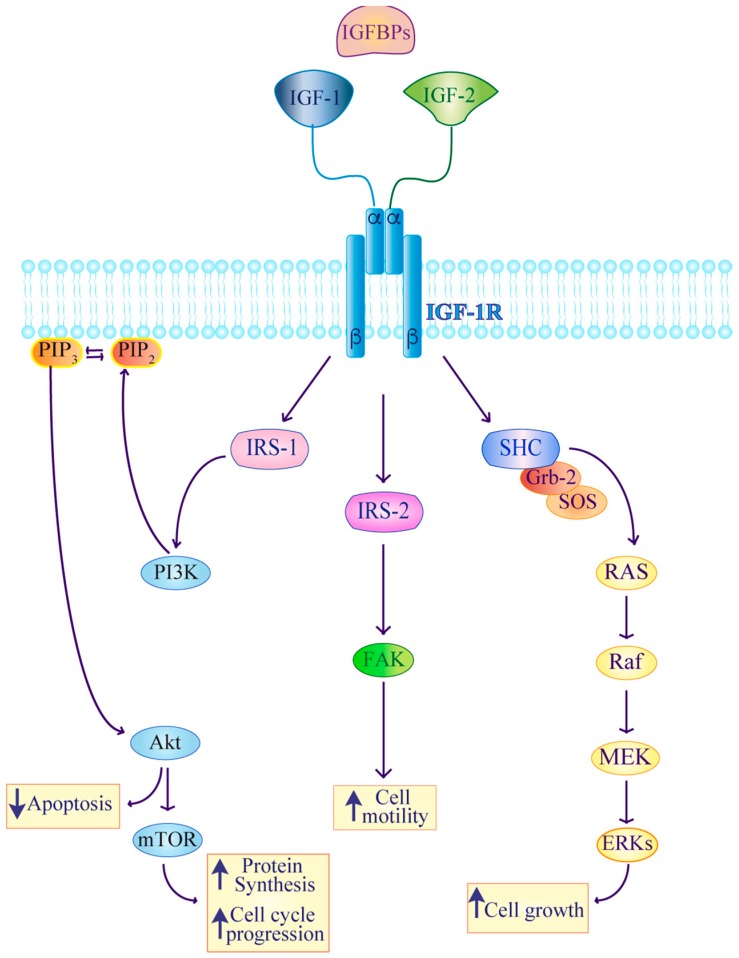
Schematic representation of the IGF-IR downstream signaling. Activation of IGF-IR is triggered by IGF1/2 ligands and modulated by IGFBPs. After ligand binding, IGF-IR recruits docking proteins including IRSs and SHC which induce the activation of intracellular modulators involved in: cell growth (RAS/RAF/MEK/ERK), protein synthesis, cell cycle progression (PI3K/AKT/mTOR) and cell motility [33]. Activated IGF-IR also inhibits apoptosis.

Signaling downstream of the IGF axis involves intracellular mediators contributing to cell proliferation Rat Sarcoma (RAS)/Proto-Oncogene Serine/Threonine-Protein Kinase (RAF)/Mitogen-Activated Protein Kinase inhibitors (MEK)/Extracellular Signal-Regulated Kinase (ERK), apoptosis inhibition, protein synthesis, cell cycle progression Phosphatidylinositol kinase (PI3K)/V-Akt Murine Thymoma Viral Oncogene Homolog (AKT)/Mammalian Target of Rapamycin (mTOR) and cell motility Focal Adhesion Kinase (FAK) [33,34,35]. Activation of the IGF-IR receptor by IGF ligands leads to its tyrosine phosphorylation thus generating a binding site for docking proteins such as Src Homology 2 Domain Containing Transforming Protein 1 (SHC) and Insulin receptor substrates (IRSs). In turn, SHC recruitment activates RAS/RAF/ERK pathway while IRS1 and IRS2 activate the PI3K/AKT/mTOR and FAK cascades, respectively (Figure 1) [36,37,38].

**Figure 2:**
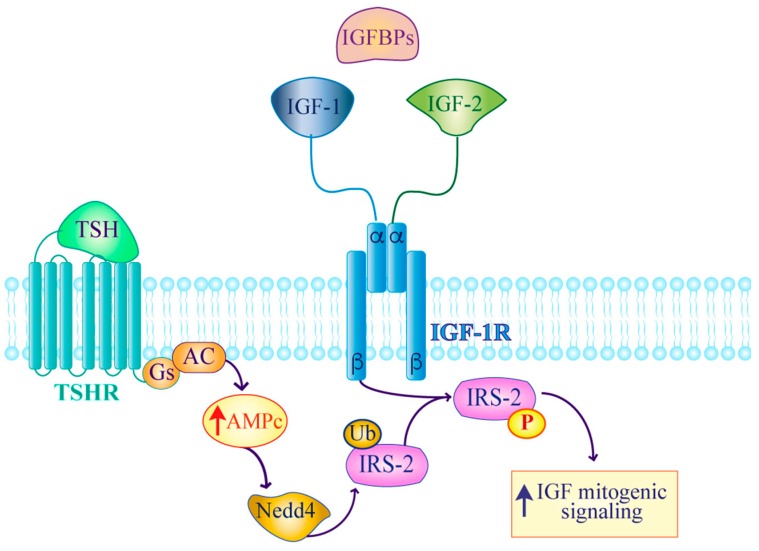
Working model depicting how the TSH/Nedd4/IRS2 axis improves IGF mitogenic activity. cAMP-stimulating agents such as TSH, promote Nedd4-dependent ubiquitination of IRS-2 thereby assembling a Nedd4-IRS-2 complex that enhances IGF-dependent mitogenic signaling. AC: Adenylyl cyclase; Gs: G Protein.

The involvement of the IGF axis in thyroid carcinoma is also related to crosstalk interactions between the IGF-IR and thyroid-stimulating hormone (TSH). In fact, the pro-tumorigenic effect of TSH is reduced in the absence of growth factors, but is increased by IGF-1 stimulation [10,39,40]. Moreover, Sarah and colleagues reported that the crosstalk between TSH and the IGF-IR through ERK and AKT pathways up-regulates NIS expression in primary tumor thyrocytes [41]. Additional data by Fukushima and colleagues indicate that cAMP-stimulating agents such as TSH induce a Nedd4-dependent ubiquitination of IRS-2 that, when associated with Nedd4, enhances IGF-dependent mitogenic effects (Figure 2) [42].

An additional layer of complexity to this already composite signaling network is represented by the well documented crosstalk between the IGF axis and several RTKs including the insulin receptor (IR), epidermal growth factor receptor (EGFR), vascular endothelial growth factor receptor (VEGFR), platelet-derived growth factor receptor (PDGFR), hepatocyte growth factor receptor (HGFR-proto-oncogene *MET*), the discoidin domain receptor tyrosine kinase 1 (DDR1) and the anaplastic lymphoma kinase 1 (ALK) [43,44,45,46,47]. The cooperation between the IGF-IR and other RTKs activate common downstream targets such as the PI3K/AKT/mTOR and RAF/MEK/ERK pathways, thus promoting tumor proliferation and inhibiting apoptosis. Moreover, although deregulation of the IGF pathway is linked to aberrant activation of the IGFRs, several mutations of its downstream targets or additional gene rearrangements may also indirectly activate the IGF axis. Obviously, these molecular events represent a potential resistance mechanism to IGF ligand/receptor inhibitors [12,48,49,50,51].

## 3. Targeting IGF Signaling in Thyroid Cancer

**Figure 3:**
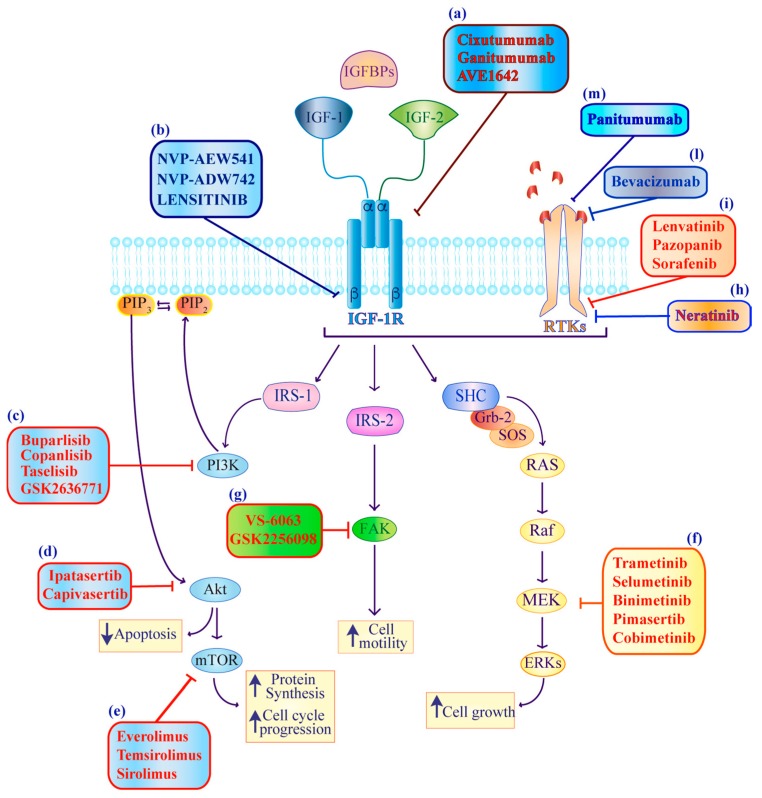
Schematic representation of direct and indirect pharmacological agents targeting the IGF axis that have been investigated in thyroid cancer. IGF-IR direct inhibitors, IGF-IR^mAbs^ (**a**) and IGF-IR^TKIs^ (**b**) reduce IGF downstream signaling. IGF-IR/RTK downstream inhibitors targeting PI3K (**c**), AKT (**d**) and mTOR (**e**) restore apoptosis while blocking protein synthesis and cell cycle progression. MEK (**f**) and FAK (**g**) inhibitors interfere with cell motility, respectively, while EGFR inhibitors (**h**), MK (multi-kinase) inhibitors (**i**) and RTK^mAbs^ (**m**) hinder the cooperation between the IGF-IR and other RTKs.

Therapeutic approaches targeting the IGF axis have been investigated in many cancers as consistent evidence suggests that IGF signaling confers resistance to several anti-tumor agents [19,52,53,54,55]. On the basis of these observations, IGF axis inhibitors have been combined with DNA damaging agents or different kinase inhibitors [43,56,57,58,59,60]. In this section, we provide an update on the current knowledge of agents that directly (*IGF-IR direct inhibitors*) or indirectly *(IGF-IR/RTKs downstream inhibitors)* interfere with the IGF axis in thyroid carcinoma (Figure 3). Specifically, we focus on findings generated in immortalized cell lines, mouse models or in clinical trials. All published or ongoing clinical trials are reported in Table 1 and Table 2.

### 3.1. IGF-IR Direct Inhibitors

Agents exerting IGF-IR inhibition include monoclonal antibodies (mAbs) targeting IGF-IR (IGF-IR^mAbs^) and tyrosine kinase inhibitors (TKIs) binding to the IGF-IR catalytic domain (IGF-IR^TKIs^) [6]. Disappointingly, clinical trials employing these agents showed modest reductions in tumor growth as multiple resistance mechanisms (an IGF2/IRA autocrine signaling loop or rising levels of circulating IGF-IR that sequesters IGF-IR inhibitors) quickly overcame their IGF-IR inhibition [54]. Thus, further preclinical and clinical studies have combined IGF-IR^mAbs^ and IGF-IR^TKIs^ with different anticancer drugs.

A comprehensive description of their possible use in the preclinical or clinical settings both, in monotherapy or in combination with additional pharmacological compounds is included below.

#### 3.1.1. IGF-IR^mAbs^

IGF-IR^mAbs^ block ligand–receptor interactions, causing receptor internalization and degradation and thereby quenching IGF-IR-mediated intracellular signaling. Several IGF-IR^mAbs^ have been generated and tested in different tumor types [6] but only AVE1642, cixutumumab and ganitumab were employed for the treatment of thyroid carcinomas.

##### AVE1642

A phase I study evaluated the efficacy of the combination of AVE1642 with docetaxel in a cohort of patients affected by different tumor types including one patient with thyroid carcinoma. More than 50% of subjects enrolled in this group achieved stable disease [61].

##### Cixutumumab (IMAC-A12)

Preclinical studies evaluated the efficacy of cixitumumab both in vitro and in vivo using an orthotopic mouse model of ATC [82]. In this study, cixutumumab decreased IGF-IR phosphorylation in a dose dependent manner. However, this inhibition only translated in a weak reduction of cell proliferation. Interestingly, combining cixutumumab with irinotecan induced cell death in vitro and strongly reduced tumor volume in the mouse model, improving survival rates compared to irinotecan alone. Following these experimental findings, two clinical trials investigated the combination of cixutumumab and different anticancer drugs in patients with thyroid carcinoma. In a phase I trial (NCT01061749), the association of cixutumumab with the MEK1/2 inhibitor selumetinib, improved time to tumor progression [62], while a nonrandomized open label phase I trial (NCT01204476) tested the association of cixutumumab with the mTOR inhibitor everolimus and octreotide in several tumor types including medullary thyroid carcinoma. To date, no results have been posted on this study.

##### Ganitumab (AMG-479)

A phase Ib basket trial investigated dual treatment with ganitumab and sorafenib or panitumumab in two patients with thyroid carcinoma [63]. In this study, the association of ganitumab with sorafenib decreased tumor size, while the combination with panitumumab only reduced tumor growth. In both patients, the best clinical response detected was disease stability.

Despite the attractive role of IGF-IR^mAbs^ as anticancer agents, an important implication concerning their adverse events profile (AEs) must be considered. These pitfalls were reported in different clinical studies and include severe AEs such as early fatal toxicities [83], hyper- or hypoglycemia, immune system impairment and cardiotoxicity [84]. Hence, when IGF-IR^mAbs^ as used as anticancer agents, it is essential apply the correct combination with other anticancer drugs in order to reduce the AEs.

#### 3.1.2. IGF-IR^TKIs^

IGF-IR^TKIs^ are small molecules that bind and functionally inhibit the IGF-IR catalytic domain. Currently, only results with linsitinib, NVP-ADW742, and NVP-AEW541 have been reported.

##### Linsitinib

The biological cooperation between TSH and IGF-IR was investigated in primary human thyrocytes where linsitinib inhibited TSH-mediated NIS stimulation that requires ERK and/or AKT signaling. These data highlight the importance of the TSH/IGF-IR crosstalk in thyroid cancer [41].

##### NVP-ADW742

Constantine and colleagues evaluated the efficacy of NVP-ADW742 against multiple tumor cell types including thyroid carcinoma cell lines. They observed that NVP-ADW742 reduces IGF-IR phosphorylation after IGF-I stimulation and inhibits cell proliferation [85].

##### NVP-AEW541

WRO thyroid cancer cells genetically modified to express the IGF-IR were implanted in thyroid glands of athymic mice and exposed to NVP-AEW541 or irinotecan, used as a reference treatment. NVP-AEW541 inhibited tumor growth more potently than irinotecan. Furthermore, NVP-AEW541 also reduced tumor angiogenesis, suggesting that IGF-IR modulates thyroid tumor microenvironment [86].

### 3.2. Inhibitors Targeting Downstream Signaling Mediators Shared by IGF-IR/RTKs (IGF-IR/RTKs Downstream Inhibitors)

A further therapeutic strategy examined in thyroid carcinomas relies on agents that interfere with the complex downstream signaling generated by the crosstalk between IGF-IR and other RTKs [16,45,51,87,88]. Preclinical and/or clinical results demonstrate that PI3K, AKT, mTOR, MEK and FAK inhibitors (i), alone or in combination, display therapeutic potential against thyroid carcinoma cells.

#### 3.2.1. PI3Ki/AKTi/mTORi

Aberrant activation of PI3K/AKT/mTOR signaling heavily contributes to thyroid tumorigenesis [89,90,91]. Hence, these IGF signaling mediators have been extensively investigated as possible therapeutic targets for thyroid cancer treatment [92,93]. A synopsis of the data available to date is summarized below.

##### Buparlisib (PI3Ki)

A phase I and a phase II clinical trial enrolling patients with refractory FTC and PDTC failed to detect reductions in tumor growth after buparlisib treatment [64,65]. These observations suggested that the triple combination of a PI3Ki, an AKTi and a MEKi might be more successful. Indeed, a trial with a PI3Ki (copanlisib, taselisib and GSK2636771) combined with an AKTi (capivasertib) and a MEKi (trametinib or binimetinib) is currently ongoing (NCT02465060).

##### Ipatasertib (AKTi)

A phase I study tested the drug’s efficacy in different tumor types including one patient with thyroid carcinoma. No results have yet been reported [66].

##### Everolimus (mTORi)

This mTOR inhibitor generated contrasting results when employed on thyroid tumors as efficacy was mostly dependent on tumor type and stage. Indeed, while low response rates were reported in patients with locally advanced or metastatic thyroid cancer [77], everolimus displayed antitumor activity in patients with advanced FTC [80] or MTC [78] and induced disease stability in an ATC case series [75]. Finally, the combination of everolimus and cisplatin [76] or sorafenib [79] showed clinical efficacy both in DTCs and MTCs.

##### Sirolimus (mTORi)

A retrospective study evaluated the efficacy of sirolimus and cyclophosphamide in patients with advanced differentiated thyroid carcinoma. The association was well tolerated, but no differences in progression-free survival were detected between patients in the experimental arm and those allocated to the control group (receiving standard of care for disease progression) [94].

##### Temsirolimus (mTORi)

The ability of temsirolimus to interfere with thyroid tumor growth was described in association with anti-angiogenic agent (trebaninib) or multikinase inhibitor (sorafenib). Specifically, a phase I trial enrolling patients with different tumor types including one with thyroid carcinoma found that the association of temsirolimus and trebaninib induced a partial response [81]. A more potent inhibition of thyroid cancer growth was achieved by combining temsirolimus and sorafenib. In detail, 36 patients affected by metastatic radioiodine-refractory thyroid cancer received the two drugs attaining stable disease in 58% of cases, a 1-year progression-free survival rate of 30.5% and an overall survival of 24.6 months [79]. Moreover, an open label phase I trial is ongoing to evaluate the efficacy of temsirolimus associated with bevacizumab or vinorelbine in patients with advanced thyroid cancer (NCT01552434 and NCT01155258).

#### 3.2.2. MEKi

Aberrant activation of intracellular signaling involving the MEK pathway contributes to thyroid tumorigenesis since MEK inhibition favors differentiation of radioiodine-refractory DTCs by restoring NIS expression [86,95,96]. Hence, a number of specific MEKi have been evaluated in preclinical and clinical studies including several samples/patients diagnosed with thyroid cancer [97,98,99].

##### Binimetinib and Pimasertib

Two phase I studies investigated the efficacy of binimetinib and the association of pimasertib with temsirolimus in patients with advanced solid tumors including thyroid cancer. However, no data have yet been reported with the exception of a case report describing a patient receiving binimetinib that achieved a partial response (tumor shrinkage >30%) [69,70].

##### Selumetinib

In a clinical trial (NCT009700359) enrolling 24 patients with thyroid cancer refractory to radioiodine, selumetinib generated clinically meaningful increases in iodine uptake [100]. This important observation, spurred an additional study that is evaluating Iodine-131 combined with selumetinib (NTC02393690). In a phase I clinical trial, Infante and colleagues reported a series of patients with different tumor types (two thyroid carcinomas) exposed to the association of selumetinib and temsirolimus. The entire cohort displayed stable disease status [71]. Finally, a phase II study that accrued 39 patients with radioiodine-refractory PTCs demonstrated that selumetinib was ineffective in patients with the *B-RAF*^V600E^ mutation [72].

##### Trametinib

This compound is an allosteric MEK inhibitor with documented anti-tumor activity against thyroid cancer. A phase I dose-escalating basket trial evaluated trametinib efficacy in five patients with thyroid carcinomas. All patients achieved stable disease or partial responses [73]. A phase IB trial assessed the combination of trametinib and everolimus in 67 patients with advanced solid tumors (one diagnosed with thyroid cancer), but the response achieved by this patient has not been reported [74]. Several additional clinical studies combining trametinib with other anticancer drugs in thyroid cancer are presently ongoing.

#### 3.2.3. FAKi

Although both in vitro and in vivo models have demonstrated an active role for FAK in thyroid tumorigenesis [16,101,102,103,104], no meaningful data have been reported in thyroid cancer patients. Two phase-I studies employing the FAK inhibitors VS-6063 and GSK2256098 in three patients with advanced thyroid cancer failed to describe any clinically significant results. This observation may be explained by the observation that blocking FAK prevents tumor invasion but likely doesn’t influence tumor size [67,68]. To date, no clinical trials investigating FAK inhibitors in thyroid cancer are available.

## 4. Conclusions

In the last few years, the incidence of thyroid carcinoma is increasing across multiple countries [31]. Although most thyroid tumors are DTCs with good prognosis, PDTCs and ATCs display unfavorable outcomes requiring new therapeutic options [51].

Increased understanding of cancer biology has led to the development of anticancer drugs targeting specific oncogenic substrates such as AKT, BCR-ABL1, EGFR, MET, and the IGF-IR. Since the IGF system is often deregulated in thyroid cancer, it has been considered an attractive pharmacological target for this disease [10]. However, compounds that directly interfere with IGF-IR activity failed to demonstrate meaningful clinical activity [6]. Different hypotheses have been developed to explain their weak efficacy in thyroid carcinomas including: (i) the persistent activation of an IGF-2-IR-A autocrine loop favoring cancer progression [6,105], (ii) IGF-IR crosstalk with other RTKs [51], compensatory intracellular signaling causing loss of therapeutic efficacy [106] and (iii) molecular alterations in RTK downstream targets (e.g., RAS or B-RAF) driving cell growth regardless of IGF inhibition [33].

In this complex scenario, combined targeted regimens may overcome these resistance phenomena and improve patient response rates [21,107,108,109,110]. Thus, dual inhibition approaches using both IGF-IR and RTKs antagonists have been investigated, but these combinations have resulted in disease stability without relevant tumor shrinkage.

Among the most promising pharmacological agents targeting downstream signaling mediators shared by both IGF-IR and RTKs, the combination of anti-mTOR compounds with different kinase inhibitors has generated encouraging reductions in tumor growth [48]. MEK inhibitors could also represent a possible therapeutic approach as these drugs may restore NIS expression in patients with radioiodine-refractory thyroid carcinoma.

In summary, while multiple pharmacological approaches aimed against the IGF axis have been actively investigated for the treatment of PDTCs and ATCs, further biological studies and additional compounds and/or pharmacological strategies will be required to significantly improve the outcome of these aggressive forms of thyroid cancer.

## Figures and Tables

**Table 1:** Clinical studies with published data.

Class of Drugs	Drug Name	Published Data	Phase	Tumor Type	N°*	Regimen
IGF-IRi	AVE1642	[61]	I	Advanced Solid	1	AVE1642+Docetaxel
	Cixutumumab	[62]	I	Advanced Solid	4	Cixutumumab + Selumetinib
	Ganitumab	[63]	I	Advanced Solid	2	Ganitumab + Sorafenib or Panitumumab
PI3Ki	Buparlisib	[64]	I	Advanced Solid	1	Monotherapy
		[65]	II	FTC + PDTC	43	Monotherapy
AKTi	Ipatasertib	[66]	I	Advanced Solid	1	Monotherapy
FAKi	VS-6063	[67]	I	Advanced Solid	1	Monotherapy
	GSK2256098	[68]	I	Advanced Solid	2	Monotherapy
MEKi	Binimetinib	[69]	II	Advanced Solid	2	Monotherapy
	Pimasertib	[70]	I	Advanced Solid	1	Pimasertib+Temsirolimus
	Selumetinib	[71]	I	Advanced Solid	2	Selumetinib + Temsirolimus
		[72]	I	PTC	39	Monotherapy
	Trametinib	[73]	I	Advanced Solid	5	Monotherapy
		[74]	I	Advanced Solid	1	Trametinib + Everolimus
mToRi	Everolimus	[75]	II	ATC	5	Monotherapy
		[76]	I	Advanced Solid	7	Everolimus + Cisplatin
		[77]	II	DTC+ATC+MTC	40	Monotherapy
		[78]	II	MTC	7	Monotherapy
		[79]	II	DTC+MTC	41	Everolimus + Sorafenib
		[80]	II	DTC+ATC	28	Monotherapy
	Temsirolimus	[81]	I	Advanced Solid	1	Temsirolimus + Trebananib
		[79]	II	DTC+ATC	36	Temsirolimus + Sorafenib

N°* of thyroid cancer patients in each trial; FTC Follicular Thyroid Cancer; PDTC Poorly Differentiated Thyroid Cancer, MTC Medullary Thyroid Cancer, DTC Differentiated Thyroid Cancer, ATC Anaplastic Thyroid Cancer.

**Table 2:** Ongoing or completed yet unpublished clinical trials.

Intervention	Population	Design	Pts (n)	Primary End Point	Status	Identifier
Cixutumumab^IGF-IRmAb^ Everolimus^mTORi^ Octreotide ^somatostatin analogue^	Advanced low- or intermediate-grade neuroendocrine cancers	Nonrandomized, Open label, phase I	27 actual	DLTs, PD, PK, SP	Completed	NCT01204476
Binimetinib ^MEKi^ Capivasertib ^Akt^ Copanlisib ^PI3Ki^ Taselisib^PI3Ki^ GSK2636771^PI3Ki^ Trametinib ^MEKi^	Genetic testing-directed targeted therapy in patients with advanced refractory solid tumors, lymphomas, or multiple myeloma	Nonrandomized, Open label, phase II (molecular analysis for therapy choice)	6452 estimated	ORR	Recruiting	NCT02465060
Everolimus ^mTORi^ Sorafenib ^MKi^	Metastatic differentiated thyroid cancer progressed on Sorafenib	Nonrandomized, Open label, phase II	40 estimated	ORR, PFS	Active, not Recruiting	NCT01263951
Everolimus ^mTORi^ Sorafenib ^MKi^	Advanced thyroid cancer naive to m-TOR inhibitors or Sorafenib	Nonrandomized, Open label, phase II	41 actual	ORR	Active, not Recruiting	NCT01141309
Everolimus ^mTORi^ Pasireotide ^somatostatin analogue^	Radioiodine-refractory differentiated and medullary thyroid cancer	Randomized, Open label, phase II	42 actual	ORR	Completed	NCT01270321
Everolimus ^mTORi^	Radioiodine-refractory thyroid cancer	Nonrandomized, Open label, phase II	33 estimated	PFS	Active, not Recruiting	NCT00936858
Everolimus ^mTORi^ Sorafenib ^MKi^	Advanced radioiodine-refractory Hurthle cell thyroid cancer	Randomized, Open label, phase II	34 estimated	PFS	Recruiting	NCT02143726
Everolimus ^mTORi^ Lenvatinib ^MKi^	Metastatic differentiated thyroid cancer progressed on Lenvatinib	Nonrandomized, Open label, phase II	40 estimated	PFS	Recruiting	NCT03139747
Everolimus ^mTORi^ Pasireotide ^somatostatin analogue^	Advanced medullary thyroid cancer	Nonrandomized, Open label, phase II	19 actual	PFS	Completed	NCT01625520
Everolimus ^mTORi^	Locally advanced or metastatic thyroid cancer	Nonrandomized, Open label, phase II	40 actual	ORR	Completed	NCT01164176
Everolimus ^mTORi^ Neratinib ^EGFRi^ or Neratinib ^EGFRi^ Trametinib ^MEKi^	Advanced cancer with *EGFR* mutation/amplification, *HER2* mutation/amplification, *HER3/4* mutation or *KRAS* mutation	Nonrandomized, Open label, phase I	120 estimated	DLTs	Recruiting	NCT03065387
Everolimus ^mTORi^ Vatalanib ^VEGFi^	Advanced solid tumors	Nonrandomized, Open label, phase I	96 estimated	DLTs, SP	Completed	NCT00655655
Bevacizumab^VEGFmAbs^ Temsirolimus ^mTORi^	Advanced or metastatic malignancies or other benign diseases	Nonrandomized, Open label, phase I	216 estimated	DLTs	Recruiting	NCT01552434
Temsirolimus ^mTORi^ Vinorelbine	Unresectable or metastatic solid tumors	Nonrandomized, Open label, phase I	19 actual	DLTs, ORR	Completed	NCT01155258
Ciclophosfamide Sirolimus ^mTORi^	Metastatic, RAI-refractory, differentiated thyroid cancer	Nonrandomized, Open label, phase II	19 estimated	ORR	Recruiting	NCT03099356
Grapefruit juice Sirolimus ^mTORi^	Advanced malignancies	Nonrandomized, Open label, phase Ib	41 actual	PK	Completed	NCT00375245
Iodine I-131 Selumetinib ^MEKi^	Recurrent or metastatic thyroid cancer	Randomized, Double blind, phase II	60 estimated	ORR	Recruiting	NCT02393690
Olaparib ^PARPi^ Selumetinib ^MEKi^	Endometrial, ovarian and other solid tumors with RAS pathway alterations and ovarian tumors with resistance to PARPis	Nonrandomized, Open label, phase I	90 estimated	DLTs	Recruiting	NCT03162627
Paclitaxel Trametinib ^MEKi^	Anaplastic thyroid cancer	Nonrandomized, Open label, early phase I	12 estimated	PFS	Recruiting	NCT03085056
Dabrafenib ^BRAFi^ Trametinib ^MEKi^	Recurrent thyroid cancer	Randomized, Open label, phase II	53 actual	ORR	Active, not Recruiting	NCT01723202
Dabrafenib ^BRAFi^ Trametinib ^MEKi^	Refractory metastatic differentiated thyroid cancer with *RAS* or *BRAF*^V600E^ mutations	Nonrandomized, Open label, phase II	87 estimated	ORR	Recruiting	NCT03244956
Pazopanib ^MKi^ Trametinib ^MEKi^	Advanced solid tumors enriched for patients with differentiated thyroid cancer, soft tissue sarcoma, and cholangiocarcinoma	Nonrandomized, Open label, phase I	89 actual	DLTs, SP	Completed	NCT01438554
RAI Trametinib^MEKi^	Mutant *RAS* or wild-type *RAS*/*RAF*, RAI-refractory recurrent and/or metastatic thyroid cancer	Nonrandomized, Open label, phase II	35 estimated	PFS, ORR	Recruiting	NCT02152995
Cobimetinib^MEKi^	Differentiated, poorly differentiated and anaplastic thyroid carcinomas	Nonrandomized, Open label, phase II	50 estimated	OS	Recruiting	NCT03181100

Acronyms: Complete remission rate (CRR); Dose-limiting toxicities (DLTs); Objective response rate (ORR); Overall survival (OS); Pharmacodynamic (PD); Pharmacokinetics (PK); Progression-free survival (PFS); Safety profile (SP).

## Abbreviations

ATC	Anaplastic Thyroid Cancer
AKT	V-Akt Murine Thymoma Viral Oncogene Homolog
AKTi	V-Akt Murine Thymoma Viral Oncogene Homolog inihibitors
ALK	Anaplastic Lymphoma Kinase
DDR1	Discoidin Domain Receptor Tyrosine Kinase 1
DTC	Differentiated Thyroid Cancer
FAK	Focal Adhesion Kinase
FAKi	Focal Adhesion Kinase inhibitors
FTC	Follicular Thyroid Cancer
EGFR	Epidermal Growth Factor Receptor
ERK	Extracellular Signal-Regulated Kinase
IGF-1	Insulin-like growth ligand 1
IGF-2	Insulin-like growth ligand 2
IGF-IR	Insulin-like growth factor receptor I
IGF-IIR	Insulin-like growth factor receptor II
IGFBP	Insulin-like growth factor binding protein
IRA	Insulin receptor isoform A
IRB	Insulin receptor isoform B
IRS	Insulin receptor substrate
IGF-	Insulin-like growth factor receptor monoclonal antibodies
IR^mAbs^	Insulin-like growth factor receptor tyrosine kinase inhibitors
IGF-IR^TKIs^	Mitogen-Activated Protein Kinase
MEK	Mammalian Target of Rapamycin inhibitors
MEKi	Mitogen-Activated Protein Kinase inhibitors
MET	MET Proto-Oncogene, Receptor Tyrosine Kinase
mTOR	Mammalian Target of Rapamycin
MTC	Neural Precursor Cell Expressed, Developmentally Down-Regulated 4
NEDD4	Sodium Iodide Symporter
NIS	Platelet Derived Growth Factor Receptor
PDGFR	Poorly Differentiated Thyroid Cancer
PDTC	Phosphatidylinositol kinase
PI3K	Phosphatidylinositol kinase inhibitors
PI3Ki	Papillary Thyroid Cancer
PTC	Proto-Oncogene Serine/Threonine-Protein Kinase
RECIST	Response Evaluation Criteria in Solid Tumors
RTK	Receptor tyrosine kinase
SHC	Src Homology 2 Domain Containing) Transforming Protein 1
TSH	Thyroid Stimulating Hormone
TKI	Tyrosine Kinase Inhibitors
VEGF	Vascular Endothelial Growth Factor
VEGFR	Vascular Endothelial Growth Factor Receptor
